# Prognostic significance of CD44V6 expression in osteosarcoma: a meta-analysis

**DOI:** 10.1186/s13018-015-0328-z

**Published:** 2015-12-23

**Authors:** Yunyuan Zhang, Chunming Ding, Jing Wang, Guirong Sun, Yongxian Cao, Longqiang Xu, Lan Zhou, Xian Chen

**Affiliations:** Department of Clinical Laboratory, The Affiliated Hospital of Qingdao University, Qingdao, 266003 China; Department of Orthopaedics, Qingdao Municipal Hospital, Qingdao, 266071 China; College of Laboratory Medicine, Key Laboratory of Laboratory Medical Diagnostics designated by Chinese Ministry of Education, Chongqing Medical University, Chongqing, 400016 China; Molecular Oncology Laboratory, Department of Orthopaedic Surgery, The University of Chicago Medical Center, Chicago, IL 60637 USA

**Keywords:** CD44V6, Osteosarcoma, Prognosis, Meta-analysis

## Abstract

Numerous individual studies evaluating the relationship between CD44V6 over-expression and prognostic impact in patients with osteosarcoma (OS) have yielded in conclusive results. This meta-analysis aimed to determine the value of cell adhesion molecule CD44V6 in prognosis of OS by conducting a systematic review and meta-analysis. A comprehensive search was conducted using PubMed (medline), Embase, ISI Web of Knowledge, Springer, the Cochrane Library, Scopus, BioMed Central, ScienceDirect, Wanfang, Weipu, and China National Knowledge Internet (CNKI) databases from inception through May 26, 2015. All available articles written in English or Chinese that investigated the expression of CD44V6 and the prognosis of OS were included. The quantity of the studies was evaluated according to the critical review checklist of the Dutch Cochrane Centre proposed by MOOSE. Finally, a total of eight studies with 486 OS patients were involved and the results indicated that the positive expression of CD44V6 predicts neoplasm metastasis (RR = 1.76, 95 % CI 1.38–2.25, *p* < 0.00001), and poor survival in OS with the pooled HR of 1.53 (95 % CI 1.25–1.88, *p* < 0.0001). No significant heterogeneity was observed among all studies. In conclusion, the present meta-analysis and systematic review strongly suggest that CD44V6 over-expression is associated with overall survival rate and metastasis in OS, and may be used as a prognostic biomarker to guide the clinical therapy for OS.

**Electronic supplementary material:**

The online version of this article (doi:10.1186/s13018-015-0328-z) contains supplementary material, which is available to authorized users.

## Introduction

Osteosarcoma (OS) is the most common malignancy originating from the bone that mainly afflicts the pediatric age group and young adults [1, 2]. It is the second leading cause of cancer-related death in adolescents due to the fact that most of the patients involve in fatal metastasis. It is approximately 20–25 % of newly diagnosed patients have detectable lung-related metastasis [3, 4]. Despite recent improvement in surgical, radiotherapy and neoadjuvant chemotherapy, the long-term survival of patients with OS is still less than 50 % [5]. At present, the ability to predict the prognosis and metastasis of OS is limited because the mechanism of oncogenetic is still not fully elucidated. So far, the clinical prognostic factors were still demographics (age and sex), site, stage, tumor size, and the response to chemotherapy. Therefore, identifying prognostic markers in OS could be informative for selecting proper management. In recent years, the expression of CD44 variant isoform V6 (CD44V6) has been identified as one of the potential prognostic and metastatic biomarkers for OS.

Biomarkers are used as tools in cancer prognosis and metastasis [6]. CD44 is a kind of trans-membrane glycoprotein and the structure contains seven extracellular domains, a trans-membrane domain, and a cytoplasmic domain [7]. Due to the alternative splicing of CD44, at least 10 variants are generated during transcription [8]. CD44V6 is one of the variant isoforms (CD44V), which has been reported to regulate the extracellular matrix, promote cell motility, and suppress tumor apoptosis [9–12]. In fact, CD44V6 has been implicated in promoting tumor progression [13]. There are increased levels of CD44V6, which could serve as a prognostic marker in various solid tumors, including myxofibrosarcoma [14], gastric cancer [15], colorectal cancer [16], non-small-cell lung cancer [17], esophageal squamous cell carcinoma, [18] and hepatocellular carcinoma [19].

Many published data suggested that over-expression of CD44V6 was associated with high risk of tumor metastasis and worse survival in patients with OS. However, some other studies showed controversial results and no consensus had been reached. To investigate the relationship between the potential biomarker and the clinical outcome, we conducted a meta-analysis of all available studies relating CD44V6 expression with patients in OS.

## Methods

### Search strategy and selection criteria

The PubMed (medline), Embase, ISI Web of Knowledge, Springer, the Cochrane Library, Scopus, BioMed Central, ScienceDirect, Wanfang, Weipu, and China National Knowledge Internet (CNKI) databases were used to conduct a comprehensive search for studies that evaluated the accuracy of CD44V6 for the metastasis and prognosis of OS. In addition to the electronic databases which published between inception and May 26, 2015, we also searched the reference list of each primary study and of previous systematic reviews. The search strategy included the following keywords variably combined by “CD44V6”, “CD44 variation 6” “osteosarcoma”, and “bone tumor”.

The present meta-analysis was conducted totally following the guidelines of preferred reporting items for meta-analysis of observational studies in epidemiology group (MOOSE) and MOOSE checklist for our study is shown in Additional file 1: Table S1.

### Inclusion and exclusion criteria

Inclusion criteria: (1) measurement of CD44V6 in OS using commercial reagents; (2) pathological diagnosis (gold standard) confirmed for newly diagnosed patients with OS; (3) the studies had to provide sufficient information to construct the 2 × 2 contingency table; and (4) publications written in English or Chinese.

Exclusion criteria: (1) OS diagnosed without a biopsy and there was no clear cut-off value in the literature; (2) similar studies from the same author as well as multiple duplicate data in the different works, excluding earlier and smaller sample data; (3) animal experiments, reviews, correspondences, case reports, talks, letters, expert opinions, and editorials without original data; and (4) studies of non dichotomous CD44V6 expression levels and absence of survival outcome were excluded.

### Quality assessment

The included studies were evaluated according to the critical review checklist of the Dutch Cochrane Centre proposed by MOOSE [20]. The key points were as following: (I) enough information of the carcinoma, (II) clear description of outcome assessment, (III) clear description of study design, (IV) clear description of CD44V6 measurement, (V) clear description of cut-off of CD44V6, and (VI) sufficient period of follow-up. The studies without mentioning all these points were excluded.

### Data extraction

Two investigators (YYZ and XC) evaluated the eligibility of all retrieved studies and extracted the relevant data independently. Extracted databases were then crosschecked between the two authors to rule out any discrepancy. Data regarding the following for each included studies were extracted independently: first authors’ surname, publication year, CD44V6 assessment methods, and the cut-off definition. Corresponding authors were contacted if further information was needed. The study was excluded if no response was received after sending a reminder.

### Statistical analysis

The statistical analysis was carried out using the Review Manager (RevMan) software version 5.3 (The Nordic Cochrane Centre, The Cochrane Collaboration, Copenhagen, Denmark) and STATA 12. All these hazard ratios and 95 % confidence interval (CI) were calculated. Pooled HR was calculated using a fixed-effects model or random-effects model to evaluate the relationship between CD44V6 expression and metastasis or overall survival. I^2^ statistics were used to evaluate the between-study heterogeneity analysis in this study. The fixed-effects model was used when there was no significant heterogeneity between the included studies (I^2^ < 30 %). Sensitivity analysis was used to shows influence by any individual study. Publication bias was estimated using a funnel plot with a Begg’s linear aggression test; funnel plot asymmetry on the natural logarithm scale of the HR was measured by a linear regression.

## Results

### Eligible studies

**Fig. 1:**
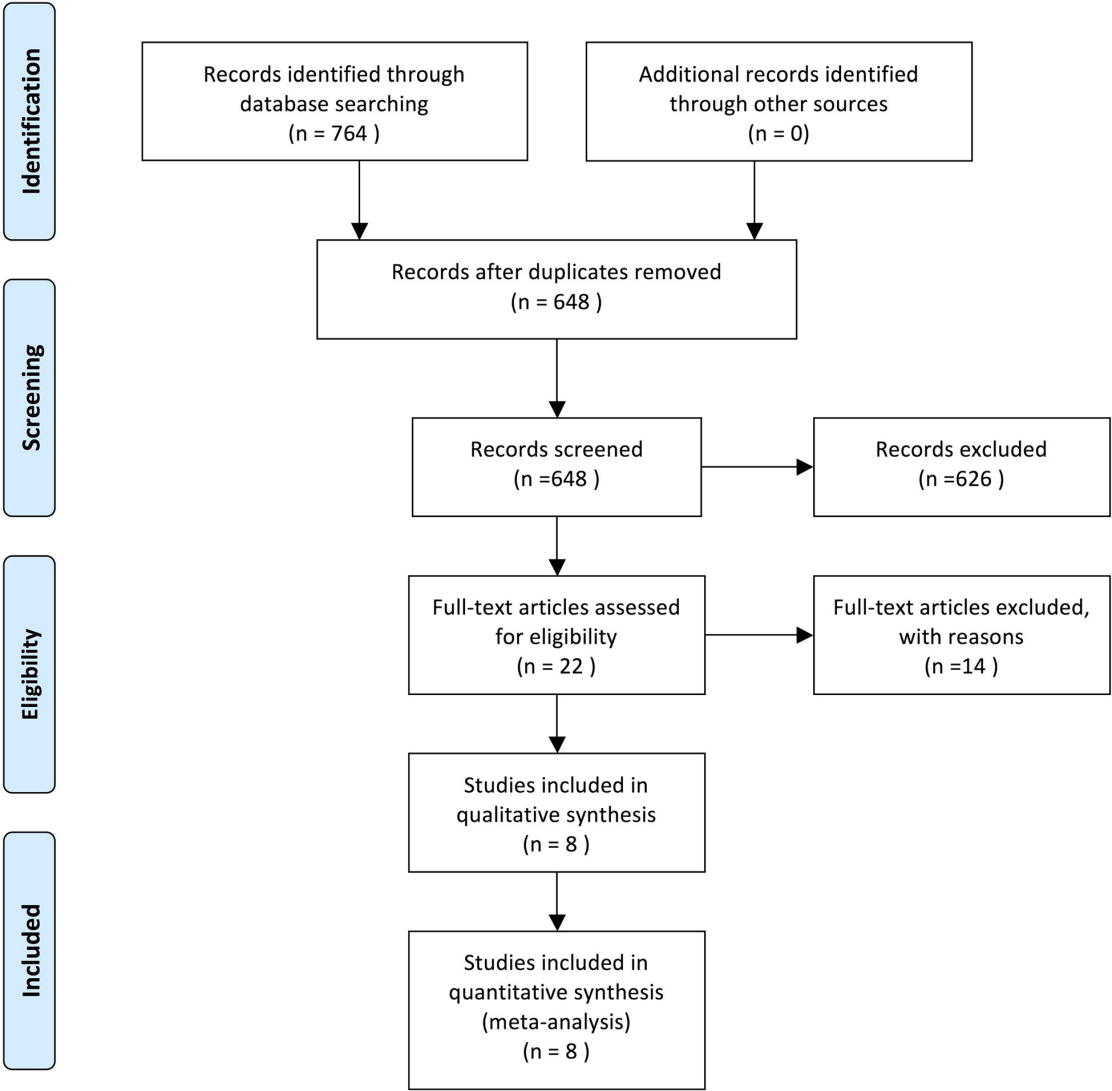
Schematic representation of the study selection

The initial search retrieved a total of 764 references that related to the metastasis or prognosis of CD44V6 in OS. After duplicated data removed, 648 articles were left. After screening titles and abstracts of identified articles, 626 articles were excluded because they were not related to the current study according to the exclusion criteria. Upon a further full text review, we excluded another 14, and 8 studies were included in our study [21–28] (Fig. 1). The main characteristics of the included studies were summarized in Tables 1 and 2. In summary, immunohistochemistry (IHC) was used for all studies to determine the expression of CD44V6. The results were judged by cut-off in two ways: percentage of positivity and immuno-reactivity (intensity) score (IRS).

**Table 1:** Characteristics of studies included in the metastasis meta-analysis

Study	Year	Age (median)	Assay kit	No. of patients	Method	CD44V6 cut-off	CD44V6 positive		CD44V6 negative	
							Metastasis	Total	Metastasis	Total
Deng et al.	2013	18.3	ZSGB-BIO	90	IHC	>6 score	38	59	12	31
Guo et al.	2007	18.6	ZSGB-BIO	49	IHC	>5 %	16	27	5	22
Hu et al.	2009	19	Santa Cruz	87	IHC	>3 score	25	45	12	42
Kim et al.	2002	17	Zymed	50	IHC	>50 %	4	26	6	24
Kuryu et al.	1999	19	R&D	39	IHC	>10 %	13	18	10	21
Li et al.	2008	23.3	Maixin	35	IHC	>0 %	14	19	3	16
Zhu et al.	2014	25	ZSGB-BIO	66	IHC	>1 score	39	56	5	10

**Table 2:** Characteristics of studies included in the 5-year survival meta-analysis

Study	Year	Age (median)	Assay kit	No. of patients	Method	CD44V6 cut-off	CD44V6 positive		CD44V6 negative	
							Metastasis	Total	Metastasis	Total
Deng et al.	2013	18.3	ZSGB-BIO	90	IHC	>6 score	56	59	20	31
Kuryu et al.	1999	19	R&D	39	IHC	>10 %	14	18	9	21
Lin et al.	2002	22	Maixin	70	IHC	>2 score	58	64	4	6
Zhu et al.	2014	25	ZSGB-BIO	66	IHC	>1 score	53	56	6	10

### Meta-analysis

**Fig. 2:**
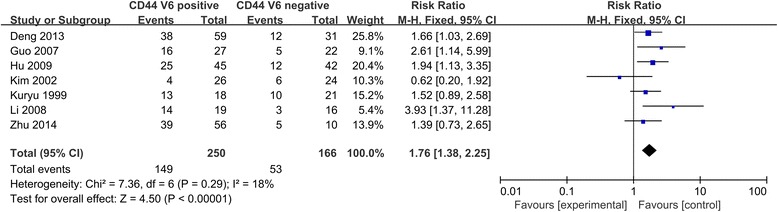
CD44V6 expression and metastasis of osteosarcoma patients

**Fig. 3:**
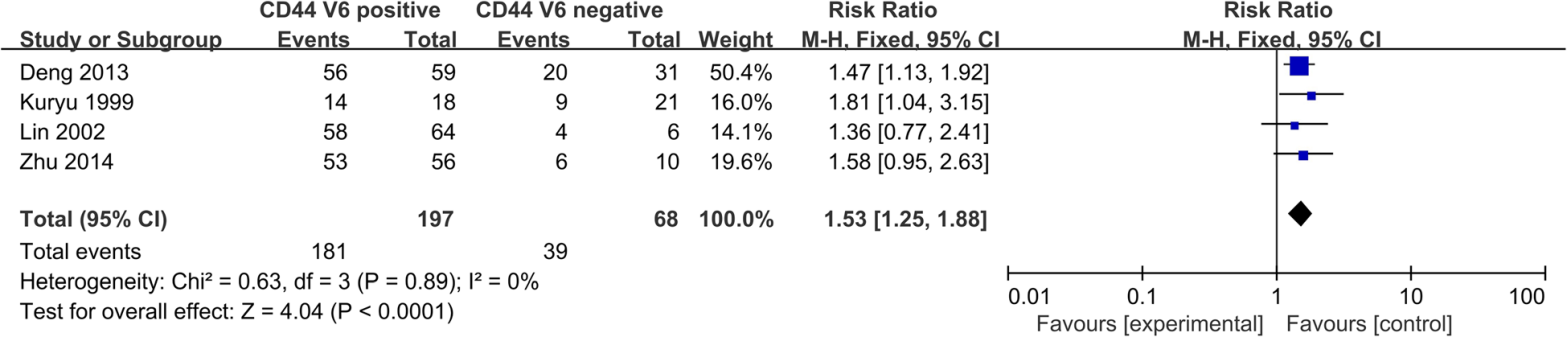
CD44V6 expression and overall survival rate of osteosarcoma patients

The intensity of relationship between the expression levels of CD44V6 and overall survival or metastasis were described as hazard ratios (HRs). For studies evaluating the overall survival and metastasis, STATA 12 and RevMan 5.3 showed there was no significant between-study heterogeneity among those studies for the overall survival of CD44V6 (I^2^ < 30 %), so the fixed-effect model was used to determine the pooled HR with corresponding 95 % CI. As shown in Figs. 2 and 3, the combined HR for all eligible studies evaluating CD44V6 over-expression on overall 5-years disease-free survival and metastasis was 1.53 (95 % CI 1.25–1.88, *p* < 0.0001, I^2^ = 0 %) and 1.76 (95 % CI 1.38–2.25, *p* < 0.00001, I^2^ = 18 %), respectively. Sensitivity analysis was used to evaluate the stability of results. No matter which study removed, the heterogeneity does not change significantly, suggesting that the results of our analysis do not overly rely on one study and the conclusions are stable. These results suggest that CD44V6 over-expression was an indicator of poor prognosis and metastasis for OS patients.

### Publication bias

Funnel plot with Revman was used to evaluate the publication bias of the literatures. The shape of the funnel plot did not reveal any evidence of obvious asymmetry (Figures not shown).

To validate the result, we also performed a Begg’s funnel plot with STATA, and no publication bias was found of both metastasis and prognosis in OS (*z* = 0.00; *p* > 0.05).

## Discussion

OS is a life threatening primary bone neoplasm that often occurs in teenagers. Disease free survival escalated from <20 % prior to the introduction of effective chemotherapy to around 60 % and overall survival to 60–70 % [29]. With 20–25 % having detectable metastases at diagnosis, OS is characterized by a high propensity for metastasis, mostly to the lungs. The overall survival rate decreases to 30 % when metastasis is detected at the time of diagnosis. Despite early detection screening protocols, improved surgical techniques, advanced radio, and chemotherapeutic regimens, limited improvements to predict the prognosis of OS patients have been achieved over the past 20 years. Therefore, the update of early prognostic biomarkers is urgently needed to adapt the proper therapy to the malignancy. Many researchers have reported that high expression of certain cell surface markers indicates bad clinical features and poor prognosis, and CD44V6 is one of the potential prognostic markers for OS.

CD44 is a trans-membrane glycoprotein, and some variant isoforms of CD44 are reportedly associated with increased invasion, metastasis, and poor prognosis [30], particularly the CD44V6 [13]. CD44V6 promoting the metastasis of cancer has been documented and the mechanism may be attributed to its interactions with various components of the extracellular matrix. Several published data have suggested that CD44V6 expression is related to metastatic potential, prognosis, and the biologic properties of human malignancies. CD44V6 was found in 69 % of prostate cancer cases [31], 26 % of non-Hodgkin’s lymphomas cases [32], 13 % of ovarian cancer cases [33], 55 % of cervical cancer [34], and 46 % of OS [35]. There are also studies showing that the presence of CD44V6 participated in the MAPK and the PI3K/Akt signaling pathways [36, 37]. Nakajima et al. suggested that CD44V6 could be an onco-protein in the bone tissue, which could be expressed in the metastasized OS [38]. The prognostic value of CD44V6 for patients with cancer has also been studied in various solid tumors. To our knowledge, no systematic meta-analysis in evaluating the prognostic values of CD44V6 and OS has been published.

Eight publications including 486 patients have been combined in the present meta-analysis, indicating a statistically significant role of CD44V6 on overall survival (HR = 1.53, 95 % CI 1.25–1.88, *p* < 0.0001) and metastasis (RR = 1.76, 95 % CI 1.38–2.25, *p* < 0.00001) in patients with OS. As a well-established, widely accepted method in medical science, immunohistochemistry (IHC) was used in all these inclusion data. We suggest that IHC should be adapted to determine the expression of CD44V6 from biopsy tissue. Our meta-analysis has its limitations and there are several issues should be considered. First, although there is no publication bias, potential publication bias may still exist. Studies with desirable results might be published more easily, which can lead to an over-estimation of overall accuracy. Second, only the studies that were published in English or Chinese were included in this meta-analysis, which could affect our results. Finally, since the rarity of primary malignant tumors of the bone, accounting for less than 0.2 % of all cancers [39], the number of included studies in this meta-analysis was only eight. It might weaken the reliability of our results. To strengthen our findings, well-designed clinical studies with larger sample size should be carried out in the future before the application of CD44V6 on the metastasis and prognosis of OS patients. Even the inherent limitations of meta-analysis is existed, this study represents a quantified synthesis of published studies and emphasizes more attention about new prognostic biomarkers on OS.

## Conclusion

In conclusion, this meta-analysis suggests that CD44V6 over-expression is associated with overall survival rate and metastasis in osteosarcoma, and may be used as a prognostic biomarker to guide the clinical therapy for osteosarcoma.

## Additional file

Additional file 1: Table S1. Presents the completed MOOSE checklist for the meta-analysis. (DOC 58 kb)
