# Supplementary material for: Prognostic significance of CD44V6 expression in osteosarcoma: a meta-analysis
Source: J Orthop Surg Res. 2015 Dec 23;10:187. doi: 10.1186/s13018-015-0328-z (PMC4690422; doi:10.1186/s13018-015-0328-z)
Supplement: Additional file 1: Table S1. — Presents the completed MOOSE checklist for the meta-analysis. (DOC 58 kb) [file 13018_2015_328_MOESM1_ESM.doc]

**Table S1**. MOOSE checklist

| **Reporting of background should include** | |
| --- | --- |
| Problem definition | Background |
| Hypothesis statement | Background |
| Description of study outcome(s) | Overall survival, metastasis value |
| Type of exposure or intervention used | Osteosarcoma |
| Type of study designs used | Meta-analysis |
| Study population | Global |
| **Reporting of search strategy should include** | |
| Qualifications of searchers (eg, librarians and investigators) | Investigator |
| Search strategy, including time period included in the synthesis and keywords | Search strategy and selection criteria |
| Effort to include all available studies, including contact with authors | We contact authors and searched reference lists and citations |
| Databases and registries searched | Methods |
| Search software used, name and version, including special features used (eg, explosion) | IE 6 |
| Use of hand searching (eg, reference lists of obtained articles) | Search strategy and selection criteria |
| List of citations located and those excluded, including justification | Flow diagram in Figure 1. |
| Method of addressing articles published in languages other than English | Search strategy and selection criteria |
| Method of handling abstracts and unpublished studies | Method |
| Description of any contact with authors | Method |
| **Reporting of methods should include** | |
| Description of relevance or appropriateness of studies assembled for assessing the hypothesis to be tested | Method |
| Rationale for the selection and coding of data (eg, sound clinical principles or convenience) | Methods |
| Documentation of how data were classified and coded (eg, multiple raters, blinding, and interrater reliability) | Methods |
| Assessment of confounding (eg, comparability of cases and controls in studies where appropriate) | Methods |
| Assessment of study quality, including blinding of quality assessors; stratification or regression on possible predictors of study results | Methods |
| Assessment of heterogeneity | Methods |
| Description of statistical methods (eg, complete description of fixed or random effects models, justification of whether the chosen models account for predictors of study results, dose-response models, or cumulative meta-analysis) in sufficient detail to be replicated | Methods |
| Provision of appropriate tables and graphics | Methods and Results |
| **Reporting of results should include** | |
| Graphic summarizing individual study estimates and overall estimate | Figure 1,2 |
| Table giving descriptive information for each study included | Table 1, 2 |
| Results of sensitivity testing (eg, subgroup analysis) | Meta-analysis |
| Indication of statistical uncertainty of findings | Discussion |
| **Reporting of discussion should include** | |
| Quantitative assessment of bias (eg, publication bias) | Results |
| Justification for exclusion (eg, exclusion of non–English-language citations) | Discussion |
| Assessment of quality of included studies | Results and discussion |
| **Reporting of conclusions should include** | |
| Consideration of alternative explanations for observed results | Discussion |
| Generalisation of the conclusions (ie, appropriate for the data presented and within the domain of the literature review) | Discussion |
| Guidelines for future research | Discussion |
| Disclosure of funding source | submission system |
